# Rapamycin improves the quality and developmental competence of *in vitro* matured oocytes in aged mice and humans

**DOI:** 10.18632/aging.204401

**Published:** 2022-11-26

**Authors:** Qiyu Yang, Juan Hu, Meng Wang, Na Guo, Liu Yang, Qingsong Xi, Lixia Zhu, Lei Jin

**Affiliations:** 1 Reproductive Medicine Center, Tongji Hospital, Tongji Medical College, Huazhong University of Science and Technology, Wuhan, China

**Keywords:** rapamycin, *in vitro* maturation, oocyte quality, aged population, DNA damage

## Abstract

Women over age 35 suffer from the inadequate number and poor quality of oocytes during assisted reproductive treatment, and making full use of the oocytes by *in vitro* maturation (IVM) is crucial. Rapamycin could improve the developmental competences of the post-maturation oocytes during *in vitro* aging, yet its effects on the IVM process of oocytes from an aged population were not clear. In this study, the immature oocytes from aged mice or older women underwent IVM with or without 10 nM rapamycin, followed by parthenogenetic activation or insemination and embryo culture. The developmental competence and quality of IVM oocytes in both groups were compared. The results showed that in aged mice, the maturation rate, activation rate, and cleavage rate of IVM oocytes were significantly elevated in the rapamycin group. Additionally, oocytes cultured with rapamycin presented decreased ROS levels, reduced chromosome aberration, and attenuated levels of γ-H2AX. During IVM of oocytes from older women, the GVBD rate, 24 h maturation rate, and 48 h maturation rate were increased in the rapamycin group, compared with those in the control group, although without significant differences. After intracytoplasmic sperm injection (ICSI) and further culture of human oocytes, the high-quality embryo rate in the rapamycin group was significantly elevated. Overall, rapamycin improved IVM outcomes of oocytes from aged mice and older women. The specific mechanism of the positive effects of rapamycin on IVM outcomes might be reducing ROS levels, mitigating DNA damage, and promoting developmental potential.

## INTRODUCTION

Delayed childbearing is an increasingly common phenomenon worldwide. The mean age of mothers at first birth has been on the rise over the last decades [1], leading to higher rates of infertility, aneuploidy, and birth defects, especially for women over age 35 [2–5]. Even though some patients have achieved live births through assisted reproductive technologies (ART), women over the age of 35 still suffer from the inadequate number and poor quality of oocytes during ART treatment, which significantly affect their therapeutic outcomes [6–8]. Therefore, making full use of the oocytes obtained (both mature and immature ones) is crucial for these older women.

Rescue-*in vitro* maturation (IVM) of immature oocytes during conventional controlled ovarian hyperstimulation (COH) cycles could partly increase the available embryos for women of all ages, especially for those with lower ovarian reserve [9–11]. Nevertheless, the embryos derived from oocytes matured *in vitro* showed lower developmental potential than those derived from oocytes matured within an ovary [12, 13], thus improving the IVM outcome is indispensable before the technology could clinically benefit older infertile women.

Rapamycin, a macrolide metabolite produced by *Streptomyces hygroscopicus*, was discovered in 1975 and applied to immunosuppressive and antifungal therapy [14, 15]. Previous studies have reported that rapamycin could affect the outcomes of oocyte IVM [16–19]. In our recent study regarding oocyte IVM in mice aged 8–10 weeks, 10 nM rapamycin was confirmed to reduce reactive oxygen species (ROS) levels and improve DNA damage repair ability, thereby reducing DNA damage levels and improving the quality and developmental potential of mouse oocytes [20]. In later experiments using oocytes from young women, we also proved that 10 nM rapamycin could significantly increase the germinal vesicle breakdown (GVBD) rate and 48 h maturation rate, improve the developmental competence of oocytes, and reduce the accumulation of DNA damage during IVM process (unpublished data). Some previous studies indicated that rapamycin could improve the developmental competences and qualities of the post-maturation oocytes during *in vitro* aging [21, 22], yet the effects of rapamycin on the IVM process of oocytes from an aged population still need to be explored.

Therefore, the current study aimed to estimate the impact of rapamycin on the quality and developmental competence of IVM oocytes from aged mice, confirm its protective effect on DNA integrity, and preliminarily verify the positive effect specifically in women over age 35.

## RESULTS

### Rapamycin improved the maturation and developmental competence of IVM oocytes from aged mice

**Figure 1:**
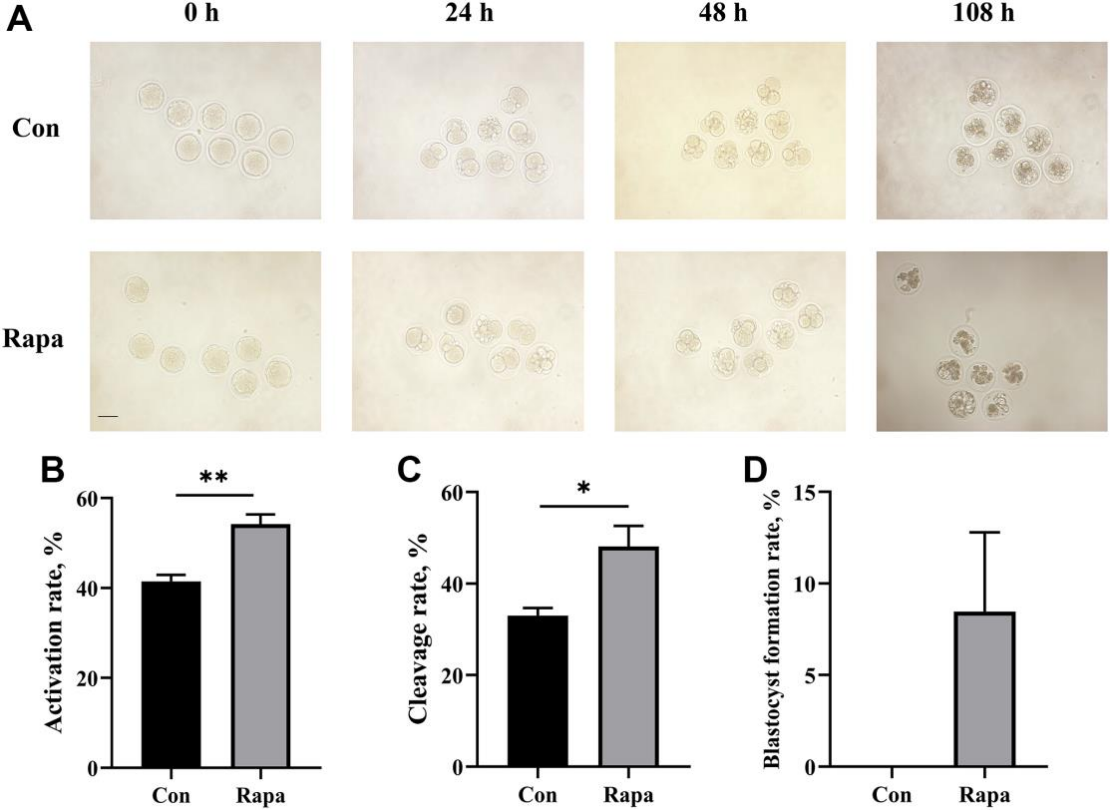
**Development of PA embryos from control and rapamycin groups.** (**A**) The status of PA embryos in both groups at 0 h, 24 h, 48 h, and 108 h of culture. Scale bar, 75 μm. The comparison of (**B**) activation rates and (**C**) cleavage rates (**D**) blastocyst formation rates of PA embryos from both groups (*n* = 59). All data were expressed as mean ± SEM from three independent experiments. Abbreviations: Con: control; Rapa: rapamycin. ^*^P < 0.05, ^**^P < 0.01.

On average, the number of immature oocytes retrieved per mouse (aged 40~48 weeks) was 18.3 ± 1.5. After IVM, the maturation rates of oocytes in the rapamycin group were significantly higher than those in the control group (85.8% ± 3.0% vs. 74.7% ± 1.9%, *P* = 0.035). Following parthenogenetic activation (PA), the activation rates, cleavage rates, and blastocyst formation rates in the rapamycin group were also increased to some extent, compared with those in the control group (activation rate: 54.2% ± 2.2% vs. 41.5% ± 1.5%, *P* = 0.008; cleavage rate: 48.1% ± 4.5% vs. 33.0% ± 1.7%, *P* = 0.034; blastocyst formation rate: 8.5% ± 4.3% vs. 0.0% ± 0.0%, *P* = 0.122), as shown in Figure 1.

### Rapamycin significantly improved the quality of IVM oocytes from aged mice

**Figure 2:**
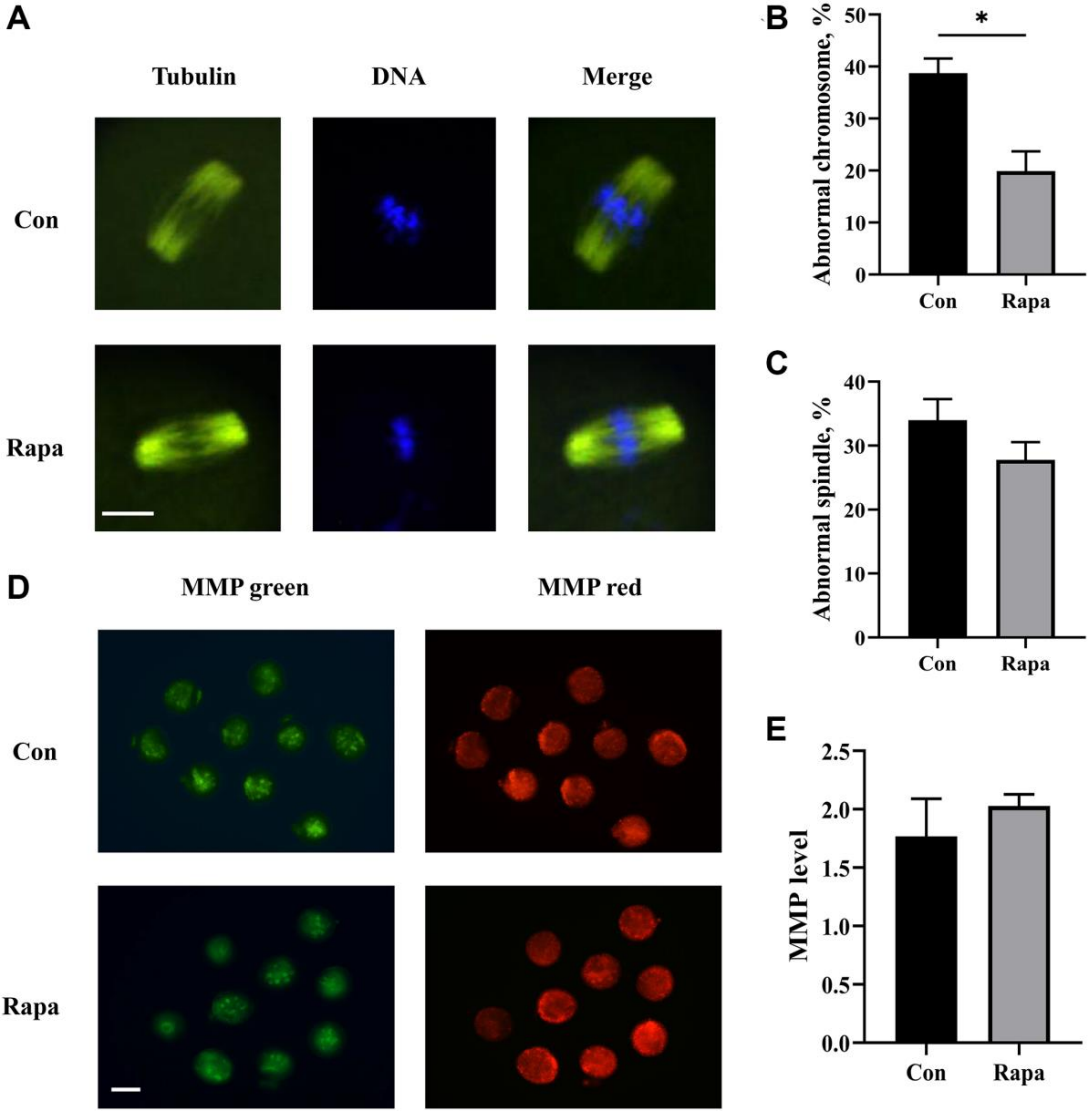
**Effects of rapamycin on the quality of IVM oocytes.** (**A**) The immunofluorescence images of spindle and chromosome of IVM oocytes from both groups. Scale bar, 25 μm. (**B**) The percentages of oocytes with chromosome aberration (*n* = 86). (**C**) The percentages of oocytes with abnormal spindle morphology (*n* = 86). (**D**) The immunofluorescence images showing JC-1 staining intensity in oocytes matured in different media. Scale bar, 75 μm. (**E**) The relative levels of MMP (red/green fluorescence intensity) in oocytes from both groups (*n* = 49). All data were expressed as mean ± SEM from three independent experiments. ^*^P < 0.05.

The quality of IVM oocytes was assessed from three aspects: spindle structure, chromosome arrangement, and mitochondrial activity. As shown in Figure 2, the rates of abnormal chromosome alignment in the oocytes of the rapamycin group were significantly reduced, compared with those in the control group (19.9% ± 3.8% vs. 38.7% ± 2.8%, *P* = 0.016). No significant difference in the abnormal rates of spindle morphology was observed between the two groups (27.8% ± 2.8% vs. 34.0% ± 3.3%, *P* = 0.224). Moreover, the levels of mitochondrial membrane potential (MMP) in the oocytes were also evaluated. The MMP levels slightly increased in oocytes of rapamycin group, compared with those of control group (2.0 ± 0.1 vs. 1.8 ± 0.3), yet without statistical significance (*P* = 0.485).

### Rapamycin markedly reduced the ROS levels and rescued DNA damage in IVM oocytes from aged mice

**Figure 3:**
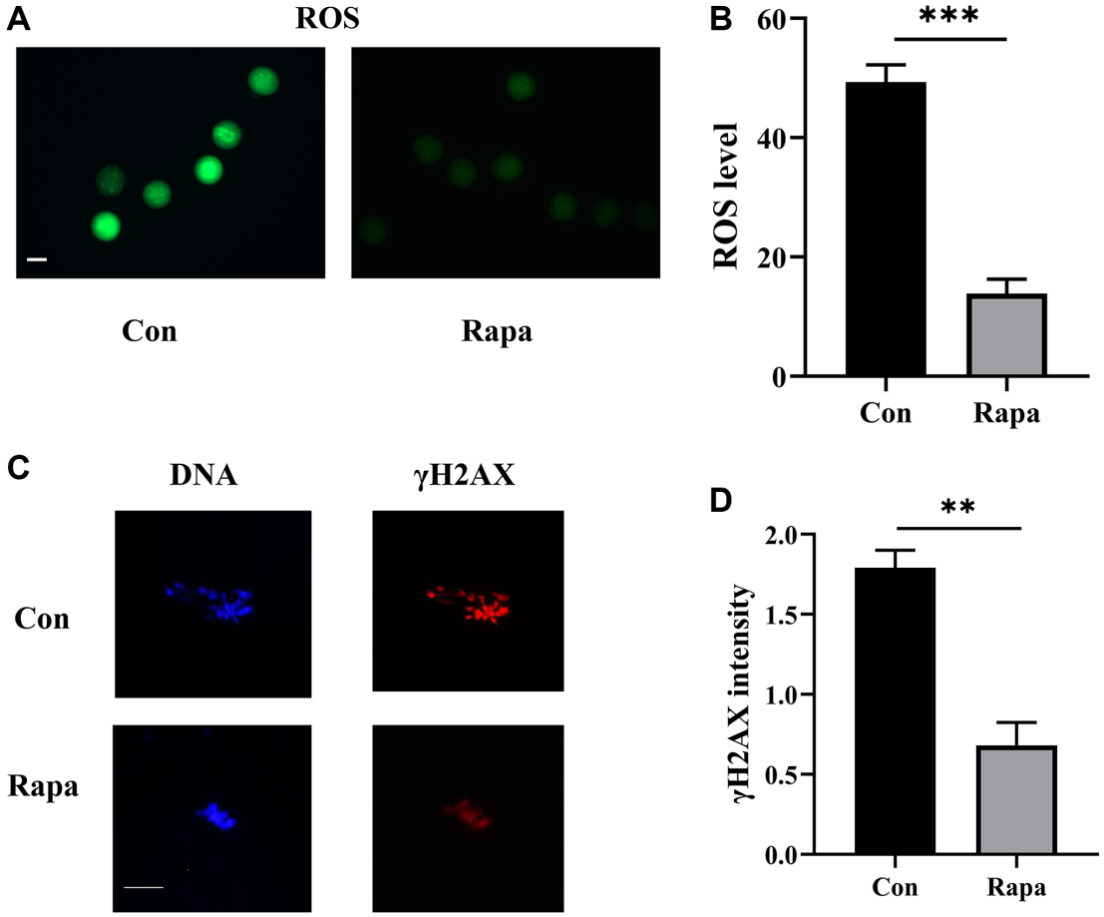
**Effects of rapamycin on the levels of ROS and γ-H2AX of IVM oocytes.** (**A**) The fluorescence images of ROS. Scale bar, 75 μm. (**B**) The levels of ROS in oocytes from both groups (fluorescence intensity value) (*n* = 43). (**C**) The fluorescence images of DNA and γ-H2AX in oocytes. Scale bar, 25 μm. (**D**) The intensity of γ-H2AX normalized to the mean DNA intensity in both groups (*n* = 29). ^**^P < 0.01; ^***^P < 0.001.

As shown in Figure 3, ROS levels in oocytes matured with rapamycin were dramatically lower than those in the control group (13.9 ± 2.4 vs. 49.4 ± 2.9, *P* < 0.001). Additionally, the DNA damage levels were assessed by examining γ-H2AX intensity in oocytes. The results showed that γ-H2AX levels were reduced significantly in the oocytes of rapamycin group (0.7 ± 0.1 vs. 1.8 ± 0.1, *P* = 0.004).

### Rapamycin promoted the developmental competence of IVM oocytes in older women

As shown in Table 1, 64 GV oocytes donated from 32 infertile couples receiving ICSI were included in the control group, and 67 GV oocytes from 40 couples were included in the rapamycin group. During IVM, 46 (71.9%) oocytes in the control group and 57 (85.1%) oocytes in the rapamycin group underwent GVBD (*P* = 0.088). The maturation rate of oocytes in the control group was 50.0% at 24 h and reached 65.6% at 48 h, and in the rapamycin group, the maturation rate was 59.7% at 24 h (*P* = 0.295) and 80.6% at 48 h (*P* = 0.075).

**Table 1:** IVM outcomes of oocytes from elderly women in both groups.

	**Control group**	**Rapamycin group**	***P* value**
Number of patients, *n*	32	40	/
Age, y	37.6 ± 0.4	38.0 ± 0.4	0.457
Number of GV oocytes	64	67	/
GVBD, *n* (%)	46 (71.9%)	57 (85.1%)	0.088
24 h maturation, *n* (%)	32 (50.0%)	40 (59.7%)	0.295
48 h maturation, *n* (%)	42 (65.6%)	54 (80.6%)	0.075
Number of ICSI oocytes	39	34	/
Normal fertilization, *n* (%)	20 (51.3%)	19 (55.9%)	0.815
High-quality embryo, *n* (%)	5 (12.8%)	12 (35.3%)	0.029

Abbreviation: *ICSI*: intracytoplasmic sperm injection.

In order to assess the developmental competence of the IVM oocytes, oocytes matured *in vitro* were collected for further insemination and continued culture. The results indicated that there was no significant difference in the normal fertilization rate (two pronuclei formation) between the rapamycin group and the control group (normal fertilization rate: 55.9% vs. 51.3%, *P* = 0.815), but the high-quality embryo (HQE) rate in the rapamycin group was significantly increased (35.3% vs. 12.8%, *P* = 0.029).

## DISCUSSION

In the current study, IVM of oocytes from aged mice and humans was performed and the effects of rapamycin on the developmental competences and qualities of IVM oocytes were investigated. The results showed that rapamycin increased the maturation rate, PA rate of IVM oocytes, and cleavage rate of the PA embryos in aged mice. Simultaneously, rapamycin also improved the chromosome arrangement, reduced ROS levels, and rescued DNA damage in the IVM oocytes. Moreover, the developmental competences of IVM oocytes from women over age 35 were also improved by rapamycin, with significantly more HQE obtained.

Combined with our previous experimental data in young mice obtained under the same experimental conditions [20], we found that the number of GV oocytes obtained per aged mouse was significantly lower than that of a young mouse, but the maturation rates after IVM were comparable between the aged and young mice, which was consistent with the results from other teams [23] and indicated the potential of IVM in increasing available oocytes for the aged population. Notably, the activation rate, cleavage rate, and blastocyst formation rate of IVM oocytes from aged mice were dramatically decreased, compared with those of young mice [20], suggesting the necessity of improving the developmental competence of IVM oocytes from aged mice.

By adding 10 nM rapamycin to the IVM medium, we observed the ameliorative effects in aged mouse oocytes including the increase in maturation rate, activation rate, and cleavage rate; and the decrease of abnormal chromosome arrangement, ROS levels, DNA damage levels, which were in accordance with our results detected in young mice [20]. We, therefore, speculated that the previous conclusion drawn in oocytes from young mice would equally apply to aged mice, i.e., rapamycin improved the quality and developmental potential of oocytes during IVM by attenuating DNA damage accumulation. Interestingly, the oocytes from older women seemed to be less sensitive to rapamycin treatment, although the upward trends in relevant developmental parameters were generally consistent with those from young women. Based on our previous study, in which approximately 85% of women were under 35 years of age, rapamycin could significantly increase the GVBD rate, 48 h maturation rate, and HQE rate of their IVM oocytes [24], while in the current study using oocytes all from older women, the elevations in GVBD rate and 48 h maturation rate were not statistically significant between groups. In addition to a certain gap in sample size between the older and young patients, the difference in results may also be attributed to the intrinsic aging-related DNA damage in oocytes from older women, which was caused by the impairment of BRCA1-related DNA double-strand break (DSB) repair [25–27]. More drugs targeting the DNA DSB repair mechanism to ameliorate aging-related DNA damage in oocytes need to be investigated in the future. Additionally, considering the labeling function of γ-H2AX on cellular hyperactivation in the absence of DNA damage [28–30], the effect of rapamycin on cellular activation and its contribution to the improvement of oocytes IVM also need to be further explored.

However, it is undeniable that in the current study regarding IVM oocytes from older women, the addition of rapamycin still increased the HQE rate from 12.8% to 35.3%, with a statistical significance. Good embryo quality, according to previous studies, was markedly related to higher implantation rate, clinical pregnancy rate, and live birth rate [31–33]. Similar reports on the impact of rapamycin on IVM oocytes in aged populations were lacking, but some studies have confirmed the positive effect of rapamycin on oocytes aged *in vitro* after maturation. In 2014, Lee et al. performed IVM in porcine cumulus-oocyte complexes (COCs) for 44 h and continued culture for 24 h or 48 h to induce oocyte aging, and different concentrations of rapamycin were added during the aging process. The results showed that 10 μM rapamycin significantly improved the developmental competence and spindle morphology of oocytes aging for 24 h, decreased ROS levels, up-regulated the expressions of a variety of development-related genes, and significantly increased the blastocyst formation rate and improved blastocyst quality after PA [21]. Similar results were obtained in another study using mouse oocytes: by adding 10 nM rapamycin during *in vitro* oocyte aging, the subsequent activation rate, cytosolic calcium level, and ROS level of oocytes were decreased, the blastocyst formation rate was increased, and the spindle morphology and chromosome arrangement were significantly improved, compared with the control group [22]. Overall, rapamycin presented a promising function in the quality improvement in mammalian aged oocytes. Combined with our previous exploration of the mechanism, we reasoned that rapamycin improved IVM outcomes mainly by enhancing the ability of oocytes to identify and repair exogenous DNA damage from excessive ROS, but has a limited effect on endogenous DNA damage at the concentration currently used.

More importantly, studies on rapamycin for aging-related diseases and prolonging lifespan have been increasing in recent years, and the anti-aging effect of rapamycin on various organs including ovaries has been demonstrated in mice [34–37]. During a period of rapamycin injection, the primordial follicles preservation, oocyte quality, and ovarian microenvironment of mice were dramatically improved. Rapamycin improved ovarian function in aged mice, with increased pregnancy rates and more healthy offspring achieved [37]. For the first time, our study demonstrated the quality-improving effect of rapamycin on oocytes matured *in vitro* in aged populations. Based on these results, we believe that rapamycin has considerable application prospects in improving oocyte quality (both *in vitro* and *in vivo*) and assisted reproductive outcomes in older women.

Overall, our results from mice to humans provided a basis and reference for clinical practice. As for the safety of rapamycin treatment, our previous study has proved that the addition of rapamycin did not cause extra aneuploidy and could provide embryos with normal karyotypes. Considering that rapamycin has been approved by the FDA for preventing organ rejection and treating cancers [38], its safety in clinical application should be relatively reassuring. In subsequent studies, we would further evaluate the effect of rapamycin intervention on animal offspring to confirm its genetic safety. Moreover, we will also further establish an optimal protocol, by appropriately increasing the concentration of rapamycin as well as expanding the sample size, to improve IVM outcomes in oocytes derived from aged populations.

In conclusion, our study preliminarily proved the effects of 10 nM rapamycin for improving IVM outcomes in oocytes from aged mice and older women. The specific mechanism might be reducing ROS levels, mitigating DNA damage, and promoting developmental potential. Further studies will be focused on the expanded sample size validation of rapamycin effects, safety assessments, and long-term impact follow-up to provide evidence for precise clinical translation and better improve IVM outcomes.

## MATERIALS AND METHODS

### Immature mice oocytes collection and *in vitro* maturation

For the animal experiment, ICR mice aged 40~48 weeks were purchased from Beijing Vital River Laboratory Animal Technology Co., Ltd. Totally, 18 aged female mice were sacrificed by cervical dislocation and 241 oocytes were obtained. All the experiments were repeated three independent times. Specifically, the bilateral ovaries were isolated, transferred into M2 medium (EasyCheck, Nanjing, China) containing IBMX (Selleck, Houston, TX, USA), and punctured with sterile needles to release immature oocytes. After being washed five times in M2 medium, the denuded GV oocytes were moved to the IVM medium (EasyCheck, Nanjing, China), with or without 10 nM rapamycin (MedChemExpress, Monmouth Junction, NJ, USA), and then cultured for 16 h at 37°C under 5% CO_2_ in humidified air. Following IVM, MII oocytes were recognized with the extrusion of the first polar body (PB1), and the maturation rates of the control group and rapamycin group were recorded and compared.

### Parthenogenetic activation and subsequent culture of IVM mice oocytes

The procedures of PA were as previously described [20]. Oocytes with two symmetrical cells each having a nucleolus at 24 h after activation were identified as successfully activated, which were subsequently cultured in CZB medium (EasyCheck, Nanjing, China) to observe the cleavage rate and blastocyst formation rate at 48 h and 108 h, respectively.

### Immunofluorescence analysis of IVM mice oocytes

The immunofluorescence staining of mouse oocytes was performed as previously described [20]. Briefly, the spindle and chromosome were stained using anti-mouse β-tubulin antibody (CST, Boston, MA, USA) and Hoechst 33258 (Servicebio, Wuhan, China), respectively. DNA damage levels were evaluated using anti-rabbit phospho-histone H2AX antibody (CST, Boston, MA, USA). For evaluation of intra-oocyte ROS and MMP, the oocytes were stained with the 2′,7′- dichlorodihydro- fluorescein diacetate (DCHF-DA) (Sigma, St. Louis, MO, USA) and MMP detection (JC-1) kit (Beyotime Biotechnology Research Institute, China), respectively. The stained oocytes were observed and photographed under a fluorescence microscope (Carl Zeiss, Batenwerburg, Germany) using fixed microscopic parameters. Image J software (NIH, Bethesda, MD, USA) were applied to measure the fluorescence intensities.

### Human oocytes IVM and ICSI

Couples undergoing ICSI owing to male-factor infertility from June 2021 to May 2022 in our center were registered, and women aged 35 and over were selected. The specific inclusion and exclusion criteria were as previously described [11]. The included patients were randomly divided into a control group and the rapamycin group. COH and oocytes retrieving were processed as the regular practice of our center [11, 39, 40]. All the oocytes with GV structure were collected and cultured in G1-plus medium (Vitrolife, Sweden) with or without 10 nM rapamycin in an incubator (37°C, 6% CO_2_, and 5% O_2_) under time-lapse monitoring. The dynamical parameters of oocyte nuclear maturation, including the time of GVBD and PB1 extrusion, were recorded. Subsequently, the matured oocytes were inseminated by ICSI, using the sperm of the patients’ husband. The fertilized oocytes from both groups were further cultured in G1-plus medium. After 16–18 h, fertilization status was checked and the presence of two pronuclei was identified as normal fertilization. The zygotes were then cultured to the cleavage stage. Embryo quality was evaluated on day 2 or day 3. HQE were defined as normally fertilized embryos with symmetrical blastomeres (no less than six) and fragmentation less than 20%, according to the Cummin’s criteria [41, 42].

### Statistical analyses

SPSS software 22.0 (IBM, USA) and Graphpad Prism 8.0 (San Diego, CA, USA) were used to analyze and visualize the data. Continuous data were presented as mean ± SEM and analyzed with Student’s *t*-test. Categorical data were expressed as number (frequency) and analyzed with Chi-Square test. Wald *P*-values were two-sided; *P* < 0.05 was considered to be statistically significant.
